# Weighted Integration of Duration Information Across Visual and Auditory Modality Is Influenced by Modality-Specific Attention

**DOI:** 10.3389/fnhum.2021.725449

**Published:** 2021-10-07

**Authors:** Hiroshi Yoshimatsu, Yuko Yotsumoto

**Affiliations:** Department of Life Sciences, The University of Tokyo, Tokyo, Japan

**Keywords:** time perception, duration perception, multisensory integration, modality-specific attention, Bayesian hierarchical model

## Abstract

We constantly integrate multiple types of information from different sensory modalities. Generally, such integration is influenced by the modality that we attend to. However, for duration perception, it has been shown that when duration information from visual and auditory modalities is integrated, the perceived duration of the visual stimulus leaned toward the duration of the auditory stimulus, irrespective of which modality was attended. In these studies, auditory dominance was assessed using visual and auditory stimuli with different durations whose timing of onset and offset would affect perception. In the present study, we aimed to investigate the effect of attention on duration integration using visual and auditory stimuli of the same duration. Since the duration of a visual flicker and auditory flutter tends to be perceived as longer than and shorter than its physical duration, respectively, we used the 10 Hz visual flicker and auditory flutter with the same onset and offset timings but different perceived durations. The participants were asked to attend either visual, auditory, or both modalities. Contrary to the attention-independent auditory dominance reported in previous studies, we found that the perceived duration of the simultaneous flicker and flutter presentation depended on which modality the participants attended. To further investigate the process of duration integration of the two modalities, we applied Bayesian hierarchical modeling, which enabled us to define a flexible model in which the multisensory duration is represented by the weighted average of each sensory modality. In addition, to examine whether auditory dominance results from the higher reliability of auditory stimuli, we applied another models to consider the stimulus reliability. These behavioral and modeling results suggest the following: (1) the perceived duration of visual and auditory stimuli is influenced by which modality the participants attended to when we control for the confounding effect of onset–offset timing of stimuli, and (2) the increase of the weight by attention affects the duration integration, even when the effect of stimulus reliability is controlled. Our models can be extended to investigate the neural basis and effects of other sensory modalities in duration integration.

## Introduction

Our perception results from receiving multimodal inputs from one’s surroundings and integrating these inputs as one. However, integrated multimodal perception may not reflect the true reality. For example, the ventriloquism effect is the illusory percept, where the perceived location of an auditory stimulus is influenced by the location of the simultaneously presented visual stimulus (Battaglia et al., 2003; Alais and Burr, 2004). Similarly, this illusion is also found in the temporal domain, as the temporal ventriloquism effect, in which the perceived timing of a visual stimulus is drawn to the timing of the auditory stimulus (Klink et al., 2011; Vidal, 2017). These illusions result from the integration of spatial or temporal information of visual and auditory modalities. Previous studies have suggested the functional importance of such multisensory integration that induced more precise perception rather than unisensory perception (Ernst and Banks, 2002; Alais and Burr, 2004; Burr et al., 2009; Hartcher-O’Brien et al., 2014; Murai and Yotsumoto, 2018). Investigating multisensory integration reveals how we perceive our world precisely in daily life.

Multimodal integration can be determined by the weighted average of the inputs of each modality (Ernst and Banks, 2002; Alais and Burr, 2004; Klink et al., 2011; Tomassini et al., 2011; Rohe and Noppeney, 2015a,b, 2018; Vercillo and Gori, 2015; Rohe et al., 2019). When visual and auditory stimuli are presented, this information is processed separately by the visual and auditory domains. The perception of visual and auditory stimuli is represented by the relative contributions of visual and auditory information. Previous studies have suggested that the weight of this integration depends on which stimulus one attends to. Vidal (2017) demonstrated that when a beep and flash pair was presented with a small temporal gap, the perceived timing of this pair was captured by the attended stimulus; when participants attended to the flash, the perceived timing of this pair was shifted to the timing of the flash, and vice versa. This attentional effect on the weight of integration has been observed in various modes, such as timing or location perception (Vercillo and Gori, 2015; Vidal, 2017).

The effects of attention on multimodal integration have also been investigated in the domain of duration perception. Multiple studies demonstrated that when visual and auditory stimuli were presented with slightly different durations, the perceived duration was influenced by the duration of the auditory stimulus regardless of the attended modality (Klink et al., 2011; Heron et al., 2013; Ortega et al., 2014), except for some situation such as when the auditory stimulus is of low intensity (Ortega et al., 2014). These results suggest that, in most situations, the auditory modality dominates the visual modality in the duration integration, and attention does not affect the weights of each modality unless the auditory signal is weak.

However, since previous studies have used visual and auditory stimuli with different durations, whether auditory dominance subsists when using the same physical stimulus duration remains unclear. Klink et al. (2011) suggested that the perceived duration of a visual stimulus can be influenced not only by the duration but also by the onset and offset timing of the simultaneously presented irrelevant auditory stimulus. In other words, the auditory dominance reported earlier may have been a consequence of each modality’s different onset and offset timing, which influenced the duration integration. The studies that reported auditory dominance (Klink et al., 2011; Heron et al., 2013; Ortega et al., 2014) have not examined this possibility.

In this study, we examined whether attention affects the weight of the attended modality in the auditory–visual duration integration while controlling the physical onset–offset timings of the stimuli. It was important that the duration of the visual and the auditory stimuli were perceptually different, while the onset and offset timings of the stimuli were the same. One solution to achieve this was to use the 10 Hz visual flicker and the 10 Hz auditory flutter: The visual flicker at around 10 Hz tends to be perceived as longer than the actual duration (Kanai et al., 2006; Herbst et al., 2013; Hashimoto and Yotsumoto, 2015, 2018; Yuasa and Yotsumoto, 2015), while the auditory flutter at around 10 Hz tends to be perceived as shorter than the actual duration (Droit-Volet, 2010; Yuasa and Yotsumoto, 2015). Thus, using the simultaneous presentation of the 10 Hz visual flicker and auditory flutter, we can investigate the attentional effects on the perceived duration while assuring physically the same onset and offset timings but perceptually different durations.

In the task, we asked participants to attend only to the visual stimulus, only the auditory stimulus, or both visual and auditory stimuli. We hypothesized that the weight of the perceived duration of each stimulus was affected by the modality to which the participants attended. For example, the perceived duration of a visual flicker tends to be longer than the actual duration (Yuasa and Yotsumoto, 2015). Therefore, we predicted that when the participants attended to the visual flicker, the weights on the flicker would increase, resulting in the simultaneous presentation of flicker, and flutter stimuli would be perceived longer. In contrast, the perceived duration of auditory flutter tends to be shorter (Yuasa and Yotsumoto, 2015). Therefore, we predicted that when the participants attended to the auditory flutter, the weights on the flutter would increase, resulting in the simultaneous presentation of flicker and flutter stimuli to be perceived shorter.

Moreover, we applied Bayesian hierarchical modeling to represent the duration integration process. Previous studies investigated the attentional effect on duration integration by a simple comparison of the perceived duration of visual and auditory stimuli across conditions without estimating the weights of each modality (Klink et al., 2011; Heron et al., 2013; Ortega et al., 2014). However, this simple comparison may not be optimal for the duration-integration across modalities. The mechanism underlying duration perception has a complex hierarchical structure with a combination of modality-specific and modality-independent processing (Klink et al., 2011; Stauffer et al., 2012; Heron et al., 2013; Murai et al., 2016). Specifically, the duration information is encoded separately in each sensory modality (Becker and Rasmussen, 2007; Murai et al., 2016; Motala et al., 2018), and then integrated across modalities (Klink et al., 2011; Stauffer et al., 2012; Heron et al., 2013; Hartcher-O’Brien et al., 2014; Ortega et al., 2014). Using Bayesian hierarchical modeling, we can define a more flexible model that matches the assumption of the hierarchical process of visual and auditory integration.

In the present study, we used models that assumed that the perceived duration of visual and auditory stimuli was the weighted average of each modality. We estimated the weight of the duration integration and examined whether the weight was influenced by the modality to the participants attended. In addition, attention-independent auditory dominance in duration integration can result from the higher reliability of auditory temporal perception (Hartcher-O’Brien et al., 2014; Vidal, 2017; Murai and Yotsumoto, 2018). To separate the effect of reliability on duration integration from the attentional effect, we created second model assuming that the weight of the duration integration is influenced not only by the attention but also by the reliabilities of each modality. Also, to further examine the role of attention on duration integration, we created the third model assuming that the weight of duration integration is influenced only by the reliabilities of each modality, not by the attention.

Finally, we compared these three models to examine which model is a more likely fit to the behavioral data; in other words, we determined whether the weight for each modality can be influenced only by attention, by both the attention and the reliability of each modality, or only by the reliability.

## Materials and Methods

### Participants

Nineteen adults (one author and 18 naïve participants, ten males and nine females) with normal or corrected-to-normal vision participated in the experiment. All participants provided written informed consent to participate in the experiment in accordance with the Declaration of Helsinki. The protocol was approved by the institutional review boards of the University of Tokyo, and all experiments were carried out in accordance with the guidelines set by the Ethics Committee of the University of Tokyo.

### Apparatus

The visual stimuli were presented on a CRT monitor (Mitsubishi Electric RDF223H, 1024 × 768 pixels, 120 Hz refresh rate). The auditory stimuli were presented through a USB digital-to-analog converter Focusrite audio interface Scarlett 2i4 1st Generation and MDR-XB500 headphones at 60 dB (Sony, Japan). All stimuli were generated using MATLAB 2018b (The MathWorks Inc., Natick, MA, United States) and Psychotoolbox (Brainard, 1996) controlled by a Mac Pro (Apple, Cupertino, CA, United States). The viewing distance was 57.3 cm, and participants were asked to stabilize their heads on a chin rest. The experiment was conducted in a dark room.

### Stimuli

We used a white disk (37.5 cd/m^2^, 5° diameter) as the visual target stimulus and simple tone (800 Hz, 60 dB) as the auditory target stimuli. Sound levels were calibrated using a WS1361 sound level meter (Wensn). To check the synchronization between visual and auditory stimuli, we recorded the timings of the visual and auditory stimuli using a photodiode and a Focusrite audio interface. The digital inputs from the photodiode and the Focusrite audio interface were received using UCA202 U-CONTROL (Behringer, Germany). We verified the difference in each timing using Audacity software (downloaded from https://www.audacityteam.org/). The onset and offset of every simple tone applied a ramp of 1 ms.

### Procedure

**FIGURE 1:**
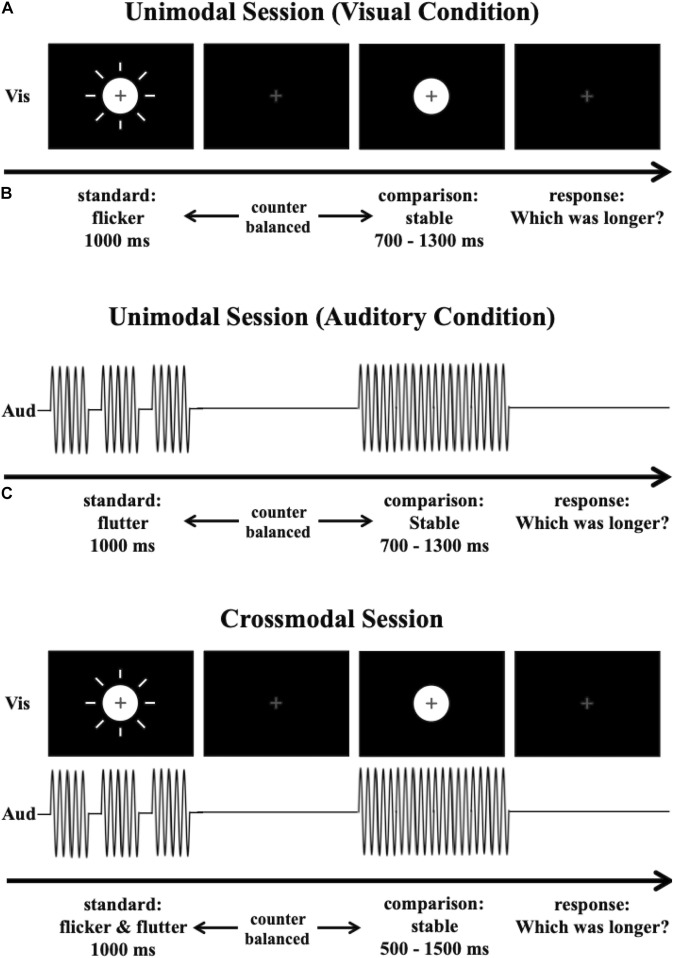
Experimental procedure. The participants were asked to compare the duration of a standard stimulus to that of a comparison stimulus. **(A)** The procedure for a unimodal session in the visual condition. **(B)** The procedure for a unimodal session in the auditory condition. **(C)** The procedure for a cross-modal session.

Figure 1 shows the experimental procedure for the duration discrimination task. In each trial, standard and comparison stimuli were presented sequentially. The interstimulus interval (ISI) was jittered between 640 and 960 ms. The duration of the standard stimulus was fixed at 1,000 ms. The duration of the comparison stimulus was randomly chosen from seven values (700, 800, 900, 1,000, 1,100, 1,200, or 1,300 ms) for each trial. The order of the standard and comparison stimuli was counterbalanced across the trials. After a standard and a comparison stimulus were presented, the participants compared the durations of the standard and the comparison stimuli and pressed a corresponding key to report which stimulus they perceived to be longer. The intertrial interval (ITI) was fixed at 800 ms. Two types of sessions (the unimodal and cross-modal sessions) were conducted on two separate days. The experimental procedure and the stimulus configurations were identical in the two sessions.

In the unimodal session, we examined the perceived duration of the visual flicker or the auditory flutter by comparing them with the perceived duration of the stable stimulus. Figures 1A,B show the experimental procedures for the unimodal session. The two modality conditions (visual and auditory) were tested in separate blocks. In this session, a total of 448 trials were divided into four blocks in which only visual stimuli were presented (visual condition, Figure 1A), and four blocks in which only auditory stimuli were presented (auditory condition, Figure 1B). Each block consisted of 56 trials. The order of the blocks was counterbalanced across participants. In the visual condition, the standard stimulus of half the trials was a visual flicker, and the standard stimulus of the other half the trials was a stable white disk. The comparison stimulus was always a stable white disk. The visual flicker consisted of a repetition of white disks for 25 ms and blanks for 75 ms (10 Hz). In the auditory condition, the standard stimulus of half the trials was an auditory flutter, and the standard stimulus of the other half the trials was a simple auditory tone. The auditory flutter consisted of a repetition of simple auditory tones for 25 ms and blanks for 75 ms.

In the cross-modal session, we examined the attentional effect on duration integration across the visual and the auditory modalities. There were three attention conditions [visual, auditory, and simultaneous visual–auditory (VA) attention]. In this session, a total of 672 trials were divided into four visual attention blocks (visual attention condition), four auditory attention blocks (auditory attention condition), and four VA attention blocks (VA attention condition). Each block consisted of 56 trials. The order of the blocks was counterbalanced across participants. In all three conditions, the visual and the auditory stimuli were presented simultaneously. In half the trials, the standard stimulus was the 10 Hz visual flicker and the 10 Hz auditory flutter presented simultaneously (flicker–flutter, Figure 1C). In the other half trials, the standard stimulus was the stable white disk and the stable auditory tone presented simultaneously (stable–stable). The comparison stimulus was always the stable white disk and the stable auditory tone presented simultaneously. At the beginning of each block, the participants were asked to attend to the specific stimuli. In the visual attention condition, participants were asked to attend to the visual stimuli while ignoring auditory stimuli, while in the auditory attention condition, participants were asked to attend to the auditory stimuli while ignoring visual stimuli. In the VA attention condition, participants were asked to attend to both visual and auditory stimuli simultaneously. Participants then compared the duration of the standard and the comparison stimuli that they attended to and answered which stimuli they perceived to be longer by pressing a key. At the middle and end of the block, we checked whether the participants attended to the stimuli that they were asked to attend. The instruction appeared on the screen for participants to press the left-, up-, or right-arrow key to report which modality they attended to in that block. The left-, up-, or right-arrow keys corresponded to the visual, visual, auditory, or auditory stimulus durations, respectively. If they attended to the stimuli that they were not asked, the data from that block were discarded, and the block with the same condition was appended to the end of the session.

### Data Analysis

In each condition, we fitted a psychometric function to the data and calculated the duration of the comparison stimulus that was subjectively equivalent to the standard stimulus of 1,000 ms [i.e., the point of subjective equivalence (PSE)]. PSE indicates the duration in which the participants responded with a 50% probability that the comparison stimulus lasted longer than the standard stimulus.

For the unimodal session, we analyzed the Bayes factor for repeated measures analysis of variance (ANOVA) using JASP free online software (v.0.10^1^, University of Amsterdam, Netherlands). We tested the hypothesis of the opposite duration distortions, where the perceived duration of the visual flicker was longer than the actual duration, while the perceived duration of the auditory flutter was shorter. The Bayes factor is the ratio of likelihood: the probability of the data given the opposite distortions between these stimuli (H_1_), divided by the probability of the data given no distortions (H_0_) (Eq. 1). Similarly, for the cross-modal session, we analyzed the Bayes factor to test the hypothesis that the perceived duration of flicker and flutter simultaneous presentation is influenced by which modality the participants attended to, the perceived duration influenced by which modality they attended to H_1_ as opposed to no influence (H_0_).

(1) $$B\,F_{10}=p\,\left(d\,a\,t\,a|H_{1}\right)/p\,\left(d\,a\,t\,a|H_{0}\right)$$

**FIGURE 2:**
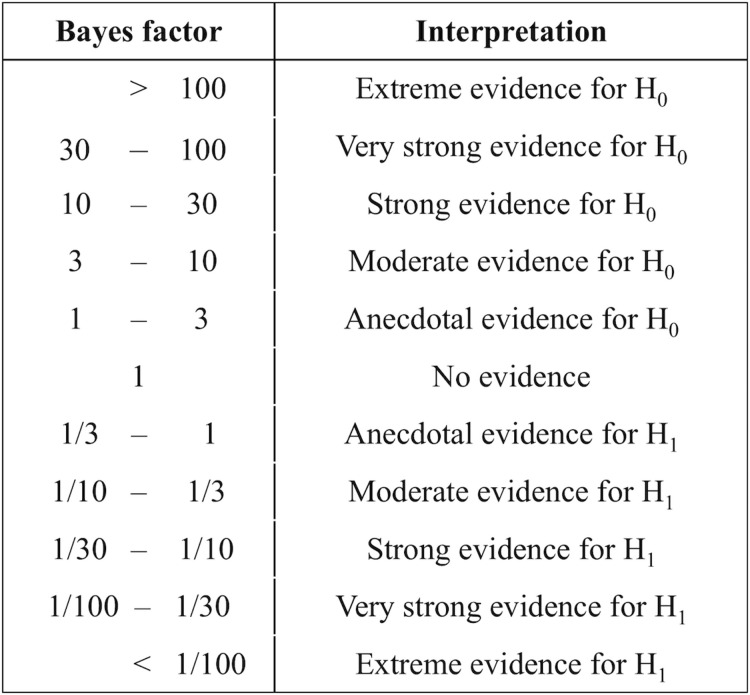
Evidence categories for the Bayes factor (Jeffreys, 1961).

The Bayes factor (*BF*_*10*_) suggests the hypothesis that our results are favored. A value of *BF*_*10*_ larger than 1 indicates that our results favor H_1_, whereas a value of *BF*_*10*_ smaller than 1 indicates that our results favor H_0_. We followed the evidence categories for the Bayes factor shown in Figure 2 to interpret *BF*_*10*_ as the strength of evidence.

**FIGURE 3:**
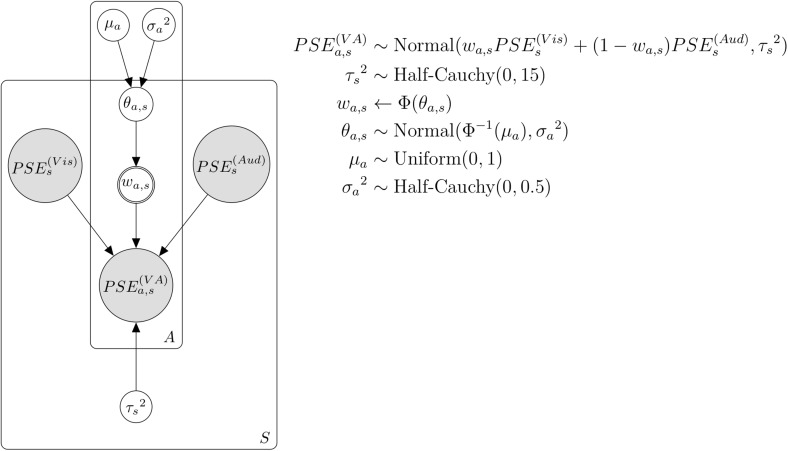
Bayesian hierarchical model with the weight by attention. *A* and *a* represent each attention condition. *S* and *s* represent each subject.

Assuming that participants perceived the duration of flicker–flutter simultaneous presentation by integrating the perceived duration of visual flicker and auditory flutter stimulus, we analyzed how much they put weights on the perceived duration of each flicker and flutter stimulus, using the Bayesian hierarchical model (Figure 3). We modeled this assumption using the following formula:

(2) $$P\,S\,E_{a,s}^{\left(V\,A\right)}∼N\,o\,r\,m\,a\,l\,\left(w_{a,s}\,P\,S\,E_{s}^{\left(V\,i\,s\right)}+\left(1-w_{a,s}\right)\,P\,S\,E_{s}^{\left(A\,u\,d\right)},\tau_{s}^{2}\right)$$

where $P\,S\,E_{a,s}^{\left(V\,A\right)}$ represents the PSE of flicker–flutter stimuli in the cross-modal session, and in each attention condition *a* (*a* = 1,2,3. each corresponds to visual, auditory, VA attention condition) and each subject *s* (*s* = 1,2,…,19. each corresponds to individual participants). $P\,S\,E_{s}^{\left(V\,i\,s\right)}$ and $P\,S\,E_{s}^{\left(A\,u\,d\right)}$ represent the PSE of the visual flicker or auditory flutter stimulus in the unimodal session, respectively, and in each subject *s*. *w*_*a,s*_ represents the weight of the perceived duration of flicker stimulus in each condition and subject. $\tau_{s}^{2}$ represents the noise parameter of the VA integration in each subject *s*. We assumed that the perceived duration of flicker–flutter stimuli in the cross-modal session followed a normal distribution with the weighted average of the perceived durations between the visual and auditory stimuli. We assumed that the variance of this distribution was integration noise. We set a weakly informative prior to this integration noise $\tau_{s}^{2}$ as follows:

(3) $$\tau_{s}^{2}∼H\,a\,l\,f-C\,a\,u\,c\,h\,y\,\left(0,15\right)$$

We assumed that *w*_*a,s*_, the weight in each condition, and the trial should be normally distributed from the group-level weight with some individual differences. We could not construct the model that *w*_*a,s*_ is directly distributed from the group-level weight because the *w*_*a,s*_ should be between 0 and 1, while the normal distribution should be between negative infinity and positive infinity. To solve this, we first transformed *w*_*a,s*_ using the probit transformation as follows:

(4) $$w_{a,s}\leftarrow \text{Φ}\,\left(\theta_{a,s}\right)$$

Then we assumed that this transformed value θ_*a*,*s*_ followed the normal distribution as follows:

(5) $$\theta_{a,s}∼N\,o\,r\,m\,a\,l\,\left({\text{Φ}}^{-1}\,\left(\mu_{a}\right),\sigma_{a}^{2}\right)$$

where μ_*a*_ represents the group-level weight in each condition and $\sigma_{a}^{2}$ represents the variance of the weights across participants. We set an uninformative prior and weakly informative prior to these parameters as follows:

(6) $$\mu_{a}\leftarrow \text{Uniform}\,\left(0,1\right)$$

(7) $$\sigma_{a}^{2}∼H\,a\,l\,f-C\,a\,u\,c\,h\,y\,\left(0,0.5\right)$$

**FIGURE 4:**
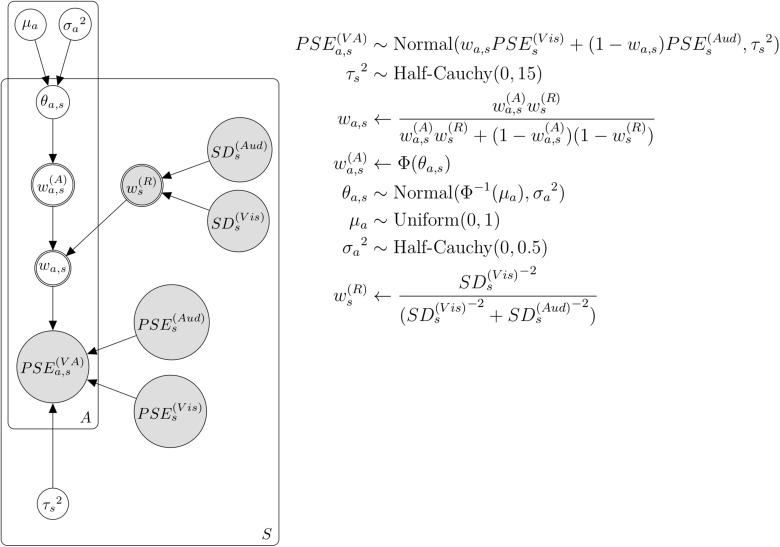
Bayesian hierarchical model with the weights by attention and reliability. *A* and *a* represent each attention condition. *S* and *s* represent each subject.

The precision of the temporal perception in the auditory modality is better than that in the visual modality (Guttman et al., 2008; Burr et al., 2009), which means higher reliability in auditory temporal perception. This difference in reliability between visual and auditory modalities can affect the weight in temporal integration (Hartcher-O’Brien et al., 2014; Vidal, 2017; Murai and Yotsumoto, 2018). We created the second model combining weight by attention and reliability (Figure 4). This model is similar to the previous model mentioned above, except for the assumption that the integration weight is influenced by attention and the reliability of each modality. We assumed weight by reliability as follows:

(8) $$w_{s}^{\left(R\right)}\leftarrow \frac{S\,D_{s}^{\left(V\,i\,s\right)-2}}{S\,D_{s}^{\left(V\,i\,s\right)-2}+S\,D_{s}^{\left(A\,u\,d\right)-2}}$$

where $w_{s}^{\left(R\right)}$ represents the weight by reliability for the perceived duration of the flicker stimulus in each condition and subject. $S\,D_{s}^{\left(V\,i\,s\right)}$ and $S\,D_{s}^{\left(A\,u\,d\right)}$ represent the reciprocal of slope parameters in psychometric function for visual stimulus or auditory stimulus, respectively, and in each condition and subject. If $S\,D_{s}^{\left(V\,i\,s\right)}$ is smaller than $S\,D_{s}^{\left(A\,u\,d\right)}$, in other words, higher reliability in visual stimuli than in auditory stimuli, the weight by reliability to flicker stimulus ($w_{s}^{\left(R\right)}$) increases. Then, we combined this weight by reliability with weight by attention as follows:

(9) $$w_{a,s}\leftarrow \frac{w_{a,s}^{\left(A\right)}\,w_{s}^{\left(R\right)}}{w_{a,s}^{\left(A\right)}\,w_{s}^{\left(R\right)}+\left(1-w_{a,s}^{\left(A\right)}\right)\,\left(1-w_{s}^{\left(R\right)}\right)}$$

where $w_{a,s}^{\left(A\right)}$ represents the weight by attention for the perceived duration of the flicker stimulus in each condition and subject, as in the previous model. *w*_*a,s*_ represents the combined weights of attention and reliability, respectively.

**FIGURE 5:**
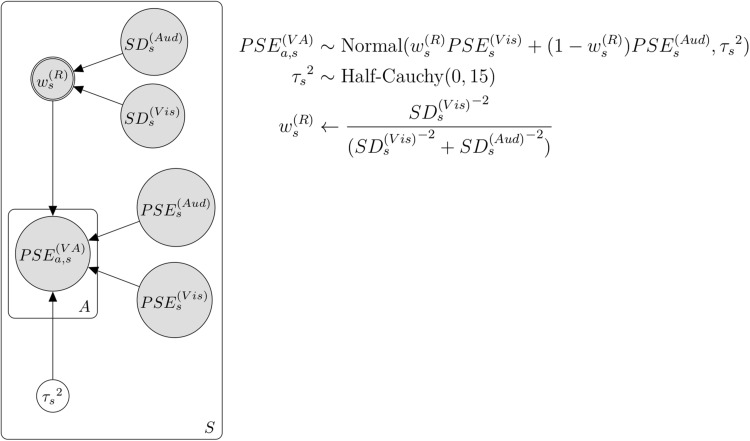
Bayesian hierarchical model with the weight by reliability. *A* and *a* represent each attention condition. *S* and *s* represent each subject.

To compare the models with and without the assumption of attentional weight, we created the third model with weight by reliability (Figure 5). This model is also similar to the previous two models mentioned above, except for the assumption that the integration weight is influenced only by the reliabilities of each modality as in Eq. 8.

These three models were created using R (version 3.6.2) and rstan (version 2.19.3; Stan Development Team, 2019). We ran four chains and sampled 30,000 samples with 15,000 warm-ups within each chain. We applied a thinning parameter of 15 to reduce the effect of autocorrelation. We checked the convergence of the obtained samples using $\hat{R}$ and the effective sample size (ESS). If $\hat{R}$ is less than 1.1, and ESS is larger than 10% of the total sample size, we can interpret that there is sufficient convergence of the obtained samples.

We analyzed Bayes factor to compare these three models using a method of bridgesampling (Gronau et al., 2017) to estimate the marginal likelihood by the package of bridgesampling [version 1.0-0 (Gronau et al., 2020)] as follows:

(10) $$B\,F_{M\,1\,M\,2}=p\,\left(d\,a\,t\,a|M_{1}\right)/p\,\left(d\,a\,t\,a|M_{2}\right)$$

where *M*_*1*_ represents the model assuming that weight is influenced only by attention, and *M*_*2*_ represents the model assuming that weight is influenced by attention and reliability. The value of *BF*_*M1M2*_ larger than 1 indicates that our results favor the model assuming the weight by the attention, whereas a BF value smaller than 1 means that our results favor the model assuming the weights by attention and reliability. In addition, we analyzed the Bayes factor comparing a model assuming weight by attention and reliability with a model assuming weight only by reliability and examined whether the assumption of attentional weight improved the model of duration integration.

## Results

**FIGURE 6:**
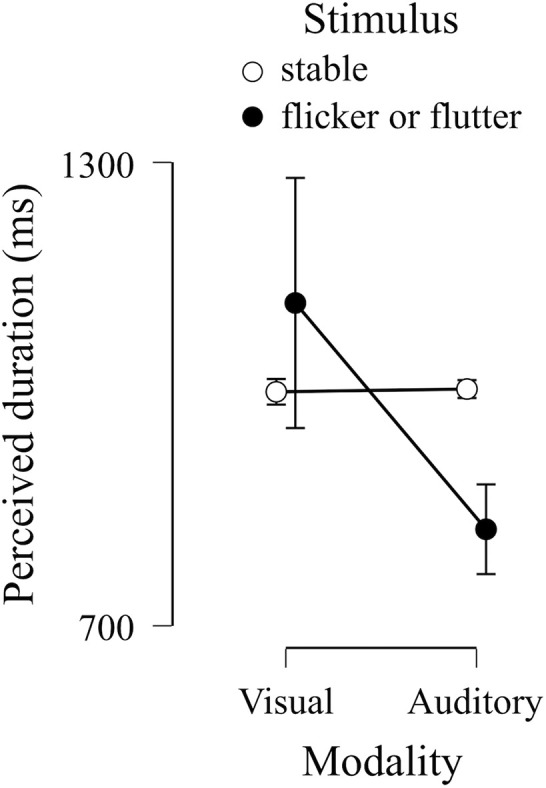
Group results of points of subjective equivalence (PSEs) in unimodal session. The dots indicate the means of PSEs in each condition. Error bars indicate a 95% credible interval.

Figure 6 shows PSEs in the unimodal session in each modality condition (visual and auditory) and each stimulus condition (stable and flicker/flutter). The PSE in the visual flicker condition was larger than that in the visual and stable condition. In comparison, the PSE in the auditory flutter condition was smaller than that in the auditory stable condition. We applied these PSEs to Bayesian repeated measures ANOVA to compare the Bayes factors between the model including the interaction effect between stimulus and modality condition, and the null model (Table 1). We found extreme evidence supporting the model, including the interaction effect between stimulus and modality condition (Bayes factor = 1.0 × 10^3^). The results suggest that visual flicker and auditory flutter induced opposite duration distortions, while the visual or auditory stable stimuli did not induce duration distortions, which was similar to that found in a previous study (Yuasa and Yotsumoto, 2015).

**TABLE 1:** Bayes factors of Bayesian repeated measures ANOVA in the unimodal session.

Models	P(M)	P(M|data)	BF_M_	BF_10_	Error %
Null model (incl. subject)	0.200	9.180e-4	0.004	1.000	
Modality + Stimulus + Modality × Stimulus	0.200	0.959	93.277	1044.544	1.592
Modality	0.200	0.030	0.125	33.039	1.008
Modality + Stimulus	0.200	0.010	0.039	10.415	2.791
Stimulus	0.200	3.107e-4	0.001	0.338	11.480

*All models include the subject. P(M) means the prior probability in each model. P(M|data) means the posterior probability given the obtained data. BF_*M*_ means the ratio of posterior odds to prior odds, while BF_10_ means the relative value of BF_*M*_ to that of null model.*

**FIGURE 7:**
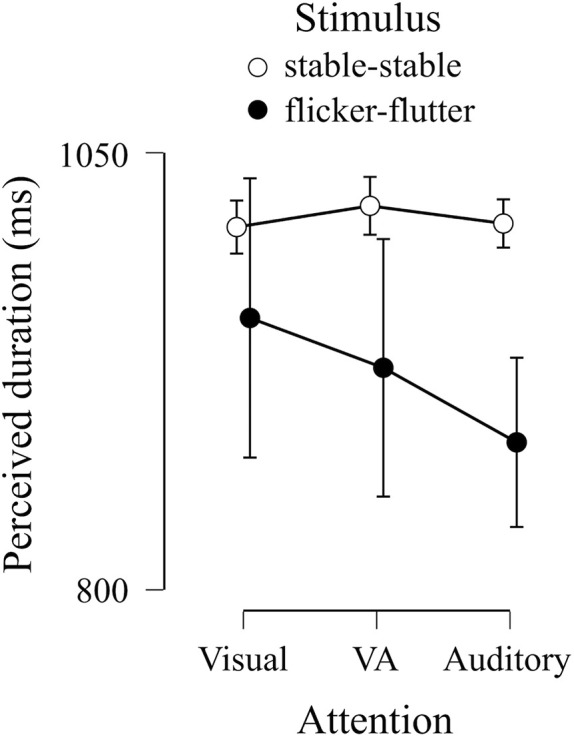
Group results of PSEs in cross-modal session. The dots indicate the means of PSEs in each condition. Error bars indicate a 95% credible interval.

Figure 7 shows PSEs in the cross-modal session in each attention condition (visual, auditory, and VA) and each stimulus condition (stable–stable and flicker–flutter). The PSEs in the flicker–flutter condition were smaller than those in the stable–stable condition. In addition, the PSEs in the stable–stable condition were almost the same across each attention condition, while in the flicker–flutter condition, the PSE while attending to the flicker tended to be larger than that found while attending to the flutter. We applied these PSEs to Bayesian repeated measures ANOVA to compare Bayes factors between each model, including the main effect or the interaction effect between stimulus and attention condition, and the null model (Table 2). We found extreme evidence supporting the model, including the main effect of stimulus condition (Bayes factors = 7.8 × 10^4^), which indicated that simultaneous flicker and flutter presentation induced duration compression in all attention conditions. We also found extreme evidence supporting the model, including the interaction effect (Bayes factor = 1.4 × 10^4^). This interaction model suggests that paying attention to one or the other modality influences the perceived duration of the flicker and flutter simultaneous presentation, but not that of the stable stimuli.

**TABLE 2:** Bayes factors of Bayesian repeated measures ANOVA in the cross-modal session.

Model comparison					

Models	P(M)	P(M|data)	BF_M_	BF_10_	Error %
Null model (incl. subject)	0.200	8.339e-6	3.336e-5	1.000	
Stimulus	0.200	0.649	7.388	77793.649	0.654
Attention + Stimulus	0.200	0.228	1.179	27304.554	2.112
Attention + Stimulus + Attention × Stimulus	0.200	0.124	0.564	14812.355	2.440
Attention	0.200	2.026e-6	8.103e-6	0.243	1.079

*All models include the subject. P(M) means the prior probability in each model. P(M|data) means the posterior probability given the obtained data. BF_*M*_ means the ratio of posterior odds to prior odds, while BF_10_ means the relative value of BF_*M*_ to that of null model.*

**FIGURE 8:**
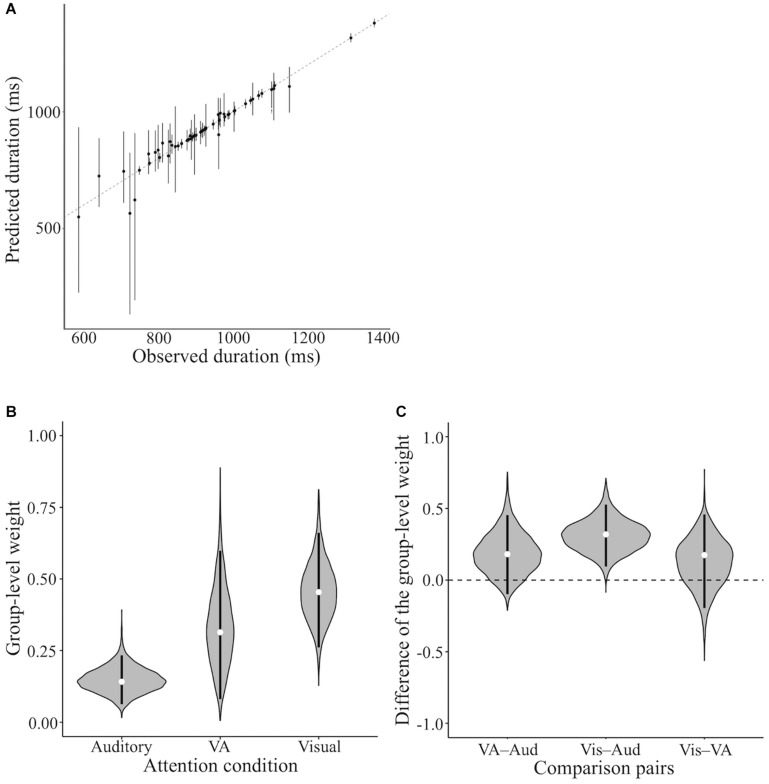
Results of the model assuming the weight by attention. **(A)** The actual and predicted PSE. The black dot indicates the mode of the simulation in each subject and condition. The black line indicates 95% highest-density-interval (HDI). The dotted line indicates the predicted PSE equal to the actual PSE. **(B)** The posterior distributions for the group-level weights (μ_*a*_) in each condition. The white dot indicates the mode of the posterior distribution. The black line indicates 95% HDI. **(C)** The posterior distributions for the difference of the group-level weights (μ_*a*_) across attention conditions. The white dot indicates the mode of the posterior distribution. The black line indicates 95% HDI. Vis, Aud, and VA represent visual, auditory, and VA attention conditions, respectively. Comparison pairs represent the difference between each attention condition (i.e., Vis–Aud represents the difference between visual and auditory attention conditions).

We assumed that the duration of integration across the visual and auditory modalities was affected by attention. We modeled this assumption using the Bayesian hierarchical model for duration integration across visual and auditory modalities (Figure 3). Figure 8A shows the relationship between the actual PSE and predicted PSE simulated by this Bayesian hierarchical model, assuming only the weight by attention. The 95% highest-density-intervals (HDI) of almost all predicted PSEs included the dotted line, which indicated that the predicted PSE was equal to the actual PSE. Thus, this model successfully predicted actual PSE. We analyzed the posterior distributions for the group-level weights by attention (μ_*a*_) in each condition (Figure 8B). The value of the weight close to 1 meant that the duration information for the visual flicker stimulus had more weights in the VA integration, whereas the value of the weight close to 0 meant that the duration information for the auditory flutter stimulus had more weights. The modes of posterior distributions of μ_*a*_in all attention conditions were smaller than 0.5, which indicated a tendency toward attention-independent auditory dominance. Although auditory dominance existed, we could also observe that the posterior distribution of μ_*a*_ was smaller in the auditory attention condition than in the VA and visual attention conditions.

To investigate whether attention to the stimulus changed the weight, we analyzed the posterior distribution for the difference in the group-level weights across attention conditions (Figure 8C). The 95% HDI of the posterior distribution far from 0 can be interpreted as the weights being different between each attention condition pair. The posterior distribution for the difference between the visual and auditory conditions was far from 0, and the 95% HDI of the difference was above 0. This indicated that the weight of the visual information was higher when attending to the visual flicker than when attending to the auditory flutter. The 95% HDIs of other comparison pairs, the comparison with VA attention condition, included 0, which meant the weights between VA attention condition and others were not different. These results suggest that the weight of duration integration across visual and auditory modalities was changed by attention; the weight of the duration information of the attended modality increased.

**FIGURE 9:**
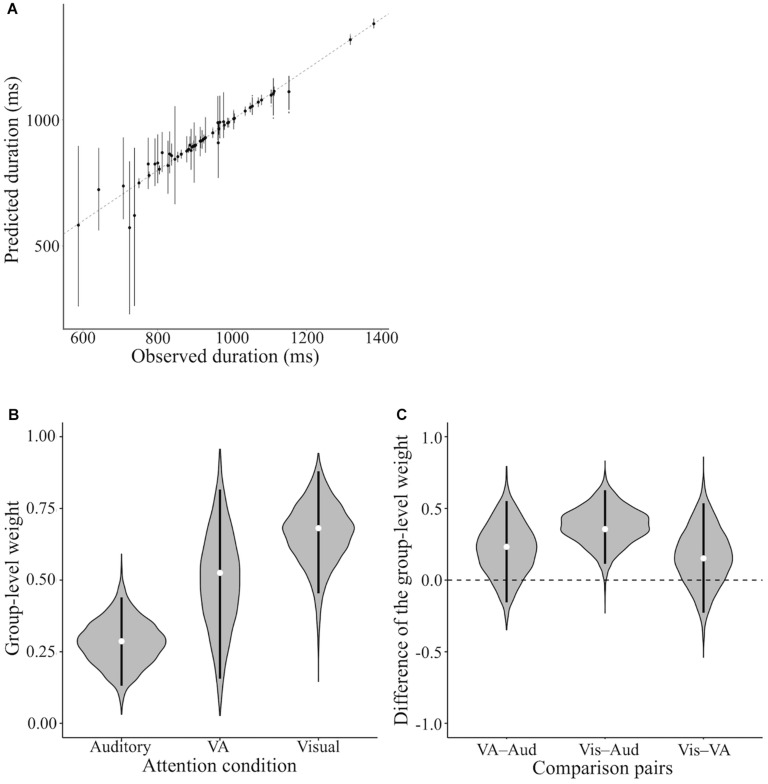
Results of the model assuming the weight by attention and reliability. **(A)** The actual and predicted PSE. The black dot indicates the mode of the simulation in each subject and condition. The black line indicates 95% HDI. The dotted line indicates the predicted PSE equal to the actual PSE. **(B)** The posterior distributions for the group-level weights (μ_*a*_) in each condition. The white dot indicates the mode of the posterior distribution. The black line indicates 95% HDI. **(C)** The posterior distributions for the difference of the group-level weights (μ_*a*_) across attention conditions. The white dot indicates the mode of the posterior distribution. The black line indicates 95% HDI. Vis, Aud, and VA represent visual, auditory, VA attention conditions, respectively. Comparison pairs represent the difference between each attention condition (i.e., Vis–Aud represents the difference between visual and auditory attention conditions).

The difference in reliability in each modality can also affect the weight of duration integration across visual and auditory stimuli. Therefore, the tendency of auditory dominance in all attention conditions may result from the higher reliability of auditory duration perception rather than visual duration perception. We modeled another Bayesian hierarchical model, which assumed that the different PSE of flicker–flutter across the attention condition results from the change in the weight by attention and the weight by reliability (Figure 4). By including the reliability of this model, we removed its effect from weight by attention. Figure 9A shows the relationship between the actual PSE and the predicted PSE simulated by this Bayesian hierarchical model, assuming the weights by attention and reliability. The 95% HDIs of almost all predicted PSEs included the dotted line, which indicated that the predicted PSE was equal to the actual PSE. Thus, this model successfully predicted the actual PSE. Figure 9B shows the posterior distributions for the group-level weights based on attention (μ_*a*_).μ_*a*_ differed across attention conditions. However, unlike the previous model, there was no tendency for auditory dominance. This difference between models implies that auditory dominance may result from the difference in reliability between each modality and that the effect of attention on the weight remains even when the effect of reliability is controlled.

We analyzed the posterior distribution for the difference in the group-level weights (μ_*a*_) across attention conditions (Figure 9C). Consistent with the previous model, the posterior distribution for only the difference between the visual and auditory conditions was above 0. Thus, the implication of this model was almost the same as that of the previous model, indicating that the weights were different when attending to visual flicker and when attending to auditory flutter. These estimates by the model assuming the weights by attention and reliability also suggest that attention influences the weight of duration integration across visual and auditory modalities.

**FIGURE 10:**
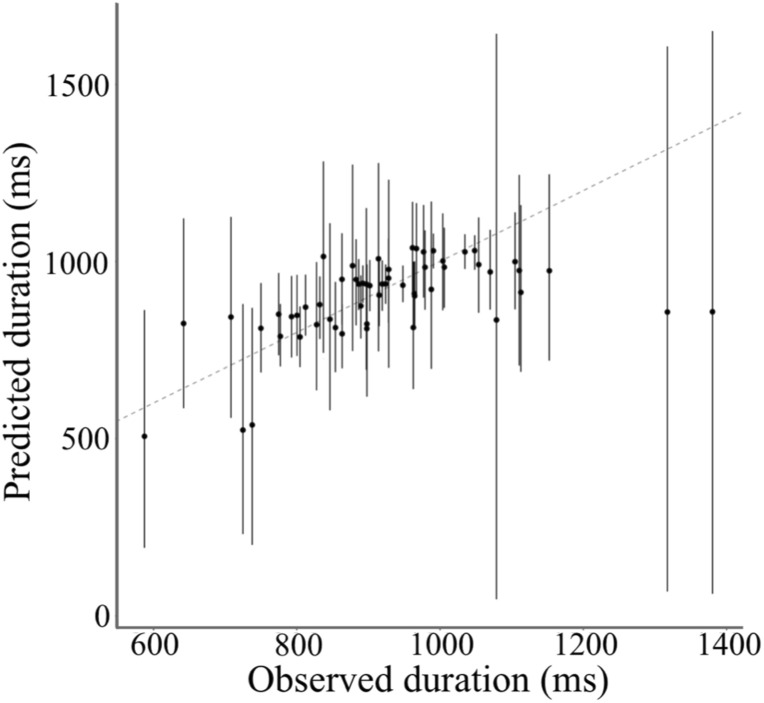
Actual and predicted PSE by the model assuming the weight by reliability. The black dot indicates the mode of the simulation in each subject and condition. The black line indicates 95% HDI. The dotted line indicates the predicted PSE equal to the actual PSE.

The previous two models demonstrated that the integration weight was influenced by which modality the participants attended to. To evaluate the assumption of attentional weight on duration integration, we modeled the third Bayesian hierarchical model, which assumed that the integration weight was influenced only by the reliabilities of each modality (Figure 5). Figure 10 shows the relationship between the actual PSE and the predicted PSE simulated by this Bayesian hierarchical model, assuming the weights by attention and reliability. The 95% HDIs of almost all predicted PSEs included the dotted line, which indicated that the predicted PSE was equal to the actual PSE. Thus, this model successfully predicted the actual PSE.

Finally, we analyzed the Bayes factor to check which model was more likely to fit the experimental data. Comparing Model 1 assuming weight by only attention, with Model 2 assuming weight by attention and reliability, we found very strong evidence supporting Model 1 (*BF*_*M*1*M*2_ = 78.658). This evidence of model comparison suggests that our results are more likely to be represented by the integration process, assuming that the weight is influenced only by attention, not by the reliability of each modality. Also, comparing Model 2 assuming weight by attention and reliability, with Model 3 assuming weight by only reliability, we found extreme evidence supporting Model 2 (*BF*_*M*2*M*3_ = 2.6794×10^24^). This evidence suggests that the assumption including attentional weight improves our models of the duration integration process.

## Discussion

We investigated whether directed attention affected the weight of the attended modality in auditory–visual duration integration. Our results showed that the perceived duration of flicker and flutter changed with attention. We then applied Bayesian hierarchical models to examine whether directed attention affects the weight of the attended modality. These models indicated that the weight on the flicker tended to be larger when the participants attended the visual flicker compared to when they attended the auditory flutter, despite the effect of the reliability in each modality.

Previous studies that reported the auditory dominance of duration integration used multiple stimuli that were presented for different durations (Klink et al., 2011; Heron et al., 2013; Ortega et al., 2014). The difference in stimulus durations led to a mismatch between the onset and offset of the stimulus presented in each modality. Hence, it is possible that the auditory dominance of duration integration partially resulted from the mismatch of the onset offset of the stimuli. To control the mismatch of the timings, we used the simultaneous presentation of a visual flicker and auditory flutter. Compared to the perceived duration of the stable stimuli, the simultaneous presentation induced a shorter perceived duration, suggesting a general dominance of the auditory modality. We further found that the perceived duration of the simultaneously presented flicker and flutter was longer when attending to the visual flicker than when attending to the auditory flutter, and when the attention was directed to both modalities, the perceived duration lay in-between them.

Our behavioral results were mostly consistent with Yuasa and Yotsumoto (2015), where they reported time dilation with the visual flicker and time compression with the auditory flutter. However, the general auditory dominance with the simultaneously presented visual flicker and the auditory flutter was only marginally significant in their study. That might be due to the statistical power and/or to the different stimulus properties used, such as brightness, loudness, pitch, and temporal frequency (Matthews et al., 2011; Matthews and Meck, 2016).

The results indicate that the perceived duration of flicker and flutter was influenced by the modality the participants attended to, consistent with the attentional effect in the audio–visual timing integration or the audio–visual spatial location integration (Vercillo and Gori, 2015; Vidal, 2017). Therefore, our results suggest that the auditory dominance reported in previous studies (Klink et al., 2011; Heron et al., 2013; Ortega et al., 2014) might have been affected by the timing of the stimulus presentation.

We also investigated the process of multisensory duration integration and estimated the weights of the attended modality using Bayesian hierarchical modeling. We used modeling to examine how attention changes the weight of each modality in duration perception. The estimations from the models support our hypothesis that the perceived duration of the attended modality was weighted more than the non-attended modality, similar to the audio–visual timing integration or the audio–visual spatial location integration (Vercillo and Gori, 2015; Vidal, 2017).

Previous studies (Hartcher-O’Brien et al., 2014; Murai and Yotsumoto, 2018) reported that the reliability of each sensory modality affects duration integration. However, how attention and reliability each contribute to duration integration remains unknown. Therefore, in the present study, we compared the two models; one model assumed that the weight of the duration integration is influenced only by the attention, while the other model assumes that the weight is influenced by the attention and reliability of each modality. We found that, regardless of reliability, attention influences duration integration.

Furthermore, we found that the estimated weights differed between models. The simple model, which did not consider the effect of reliability, revealed larger weights on the auditory flutter, independent of the attended modality. In contrast, the other model, which removed the effect of reliability, revealed no auditory dominance. These results imply that the difference in the reliability between the visual and auditory modalities may lead to the previously reported auditory dominance in duration integration (Klink et al., 2011; Heron et al., 2013; Ortega et al., 2014).

Overall, the model comparison favored the models assuming the attentional effect on duration integration over the model without assuming it. The result of the model comparison supports the influence of attention in duration integration across modalities. On the other hand, the comparison also favored the model that did consider the attention, but not reliability, over the model that considered both attention and reliability. That result was unexpected, given that reliability has been reported to influence duration integration (Hartcher-O’Brien et al., 2014; Murai and Yotsumoto, 2018). While all our models reasonably described the data, our model might not have grasped the full picture of the complexity of duration integration. For example, we did not consider the interaction between attention and reliability, while Vercillo and Gori (2015) demonstrated that attending to one modality affected the reliability of the attended modality. In addition, our model assumed the equal contribution of both attention and reliability to the duration integration, although attention may influence the integration to a greater extent than reliability, or vice versa. The purpose of the present study was not to find the best model to capture the complexity of the duration integration; rather, it was to investigate whether attention affects modality weight on duration integration while considering the influence of reliability. Thus, the present study serves as a starting point for future studies to examine more detailed processes.

Future studies can also extend our models to consider other factors that influence duration integration. For example, we could examine the integration with other senses such as tactile (Tomassini et al., 2011; Vercillo and Gori, 2015). We can also examine the influence of spatial information on integration. Temporal integration between audiovisuals becomes more difficult as the distance between each sensory input increases (Godfroy et al., 2003; Lewald and Guski, 2003). In addition, the perceived duration of the target stimulus is affected by the location of the distractor stimulus (Okajima and Yotsumoto, 2016). Based on the influences of spatial information on temporal integration, the attentional effect on multisensory duration integration may also be influenced by the spatial location of each stimulus.

In the present study, we used the 10 Hz visual flicker and auditory flutter to examine the effect of modality-specific attention on duration integration while controlling the confounding effect of the onset–offset timing of stimuli. The behavioral results demonstrated that the perceived duration of flicker and flutter was influenced by which modality the participants attended. Moreover, our modeling results supported the idea that the modality-specific attention modulated the weight of duration integration across visual and auditory modality, regardless of the reliability of each modality. Based on the effects of attention and reliability explored by our study, further studies may: (1) explore the complex influence of attention and reliability on duration integration, and (2) add other sensory and spatial information to our models.

## Data Availability Statement

The datasets presented in this study can be found in online repositories. The names of the repository/repositories and accession number(s) can be found below: https://osf.io/v3fqr/?view_only=b6b3ba978600405a949bbbd929ebbfcc.

## Ethics Statement

The studies involving human participants were reviewed and approved by Internal Review Board of the University of Tokyo. The patients/participants provided their written informed consent to participate in this study.

## Author Contributions

HY and YY designed the experiments and wrote the manuscript. HY conducted the experiments and analyzed data. Both authors contributed to the article and approved the submitted version.

## Conflict of Interest

The authors declare that the research was conducted in the absence of any commercial or financial relationships that could be construed as a potential conflict of interest.

## Publisher’s Note

All claims expressed in this article are solely those of the authors and do not necessarily represent those of their affiliated organizations, or those of the publisher, the editors and the reviewers. Any product that may be evaluated in this article, or claim that may be made by its manufacturer, is not guaranteed or endorsed by the publisher.

## Footnotes

1
https://jasp-stats.org/
